# A comparison of opioids and benzodiazepines dispensing in Australia

**DOI:** 10.1371/journal.pone.0221438

**Published:** 2019-08-19

**Authors:** M. Mofizul Islam, Dennis Wollersheim

**Affiliations:** 1 Department of Public Health, La Trobe University, Melbourne, Victoria, Australia; 2 Health Information Management, Department of Public Health, La Trobe University, Melbourne, Victoria, Australia; University of South Australia, AUSTRALIA

## Abstract

**Background:**

Inappropriate utilization of prescription opioids and benzodiazepines is a public health problem. This study examined and compared user-types and trends in dispensing of these medicines, and identified associated factors related to the duration of dispensing in Australia.

**Methods:**

A random 10% sample of unit-record data of opioids and benzodiazepines dispensed nationally during 2013–2016 was analyzed. Users were categorized into four types: single-quarter (i.e., three months), medium-episodic (dispensed 2–6 quarters), long-episodic (dispensed 7–11 quarters), chronic (dispensed 12–16 quarters). Dispensing quantity was computed in defined daily dose (DDD). Generalized multilevel ordinal models were developed to examine the factors associated with the duration of dispensing.

**Results:**

There were similarities in terms of trends of dispensing of opioids and benzodiazepines in Australia. Overall, more people were dispensed opioids than benzodiazepines. Around 52% of opioids users and 46% of benzodiazepines users were dispensed these medicines for a single quarter. However, chronic users were dispensed 60% of opioids and 50% of benzodiazepines in DDD/1000 people/day, respectively. On average, 16.6 DDD/1000 people/day of opioids and 14.2 DDD/1000 people/day of benzodiazepines were dispensed in Australia during the study period. Tasmania was dispensed the highest quantity (in DDD/1000 people/day) of these medicines, followed by South Australia and Queensland. Women compared to men, and clients of age-group 20–44, 45–64 and 65+ compared to age-group 0–19, were significantly more likely to have dispensed opioids/benzodiazepine for a relatively long duration. Clients with a history of dispensing of one of these two medicines were significantly more likely to have dispensed the other for a relatively long period.

**Conclusions:**

There were similarities in patterns of dispensing of opioids and benzodiazepines in terms of user characteristics and structural variables. Consistent use of real-time drug monitoring program and tailored intervention are recommended.

## Introduction

Opioids and benzodiazepines are central nervous system drugs, which are included in the World Health Organization’s list of essential medicines [1]. Opioids are primarily used for treating pain [2], and benzodiazepines for anxiety disorders, seizures, acute insomnia, and alcohol withdrawal [3]. However, excessive and or inappropriate prescribing and dispensing of prescription opioids and benzodiazepines may cause a range of harms including fatal overdose. Long term use of these medicines can result in physical dependence and addiction [4]. Because of their addictive properties, relatively easy availability and affordable price, sometimes these medicines are used inappropriately. In recent time, there has been a growing concern in many western countries about excessive and or inappropriate utilization of prescription opioids and benzodiazepines, as the harms associated with inappropriate use of these medicines gradually turned into a public health problem [5–8]. In the USA, for instance, inappropriate utilization of these medicines, particularly prescription opioids, became an epidemic in recent time. Although in a lesser extent than USA, inappropriate utilization of these medicines is a major public health concern in Australia [5].

Until safer and more effective treatments (e.g., abuse-deterrent formulation) for the conditions for which opioids and benzodiazepines are prescribed, “silver buckshot” rather than “silver bullets” remains the most feasible approach to address the crisis [9]. Of the many efforts under the “silver buckshot”, perhaps public health measures remain the mainstay of intervention for reducing inappropriate and or excessive utilization of these medicines. Epidemiology and surveillance capacity are core to the public health measures. Studying dispensing data and their temporal and spatial variations, socio-economic condition of local areas, and user characteristics can offer valuable information, alert atypical use and prompt action by the authorities and service providers [10,11].

Previous research on dispensing of prescription opioids in Australia mainly used aggregate data [12,13], which offers a limited scope of assessing the individual user’s characteristics to dispensing. Although few studies analyzed unit record data, they covered specific states and examined the dispensing of opioids only [14]. A clear picture of dispensing of prescription opioids and benzodiazepines and their comparison may offer valuable information on future policy and planning about public health measures around these medicines. The aim of this study was to examine and compare the dispensing of prescription opioids and benzodiazepines in Australia.

## Materials & methods

### Dataset

A 10% random sample of users who were dispensed prescription opioids and or benzodiazepines between 1 January 2013 and 31 December 2016 were collected from the Australian Government Department of Human Services. The dataset was extracted based on the date of supply and all data were fully anonymized before they were supplied. In Australia, as per the universal healthcare scheme’s Medicare subsidy type, all prescription opioids and benzodiazepines that are dispensed can be classified into following four categories: Pharmaceutical Benefits Schemes (PBS), Repatriation Pharmaceutical Benefits Scheme (RPBS), under co-payment, and private. Prescription opioids/benzodiazepines that were dispensed through private prescription were not available in this dataset. In Australia, around 7% of community prescriptions are dispensed through private prescriptions [15], and around 80% of all prescription medicines dispensed are subsidized by the PBS [16], The dataset contained a range of variables including subsidy type, individual’s sex; age; date-month-year of dispensing; generic name, form and strength; the quantity dispensed; and the local government area (LGA) of patient postcodes using 2011 Australian Bureau of Statistics concordance. Due to confidentiality concern, records for dispensing among the clients of age-group <10 years were excluded/suppressed.

This study also used population data for individual states/territories and the Index of Relative Socio-economic Disadvantage (IRSD) score of Socio-Economic Indexes for Areas (SEIFA). IRSD ranks Australian areas according to relative socio-economic disadvantage. A relatively high score indicates a lower level of disadvantage and a low score indicates a higher level of disadvantage. SEIFA is derived by the Australian Bureau of Statistics from data collected in the five-yearly national census. The term SEIFA instead of IRSD is used hereafter. All LGAs were categorized either as urban or rural by using the Australian Classification of Local Government [17].

### Analysis

This study used descriptive analysis to examine the average duration of dispensing in quarters (i.e. in three months). We used dispensing dates to compute dispensing duration. A person was identified as a user in a quarter if he/she was dispensed at least once during that quarter. Thus, each person was identified as a user for at least one and a maximum of 16 quarters. Based on the total number of individual months a user was recorded to have been dispensed these medicines, all users were then categorized into the following four types: single-quarter user (if dispensed only during one quarter); medium-episodic user (if dispensed 2–6 quarters); long-episodic user (if dispensed 7–11 quarters); and chronic user (if dispensed 12–16 quarters). For instance, an individual was identified as a chronic opioid user if she/he had been dispensed opioids at least once in each quarter and a total of 12 or more quarters. Percentages of these four types of user were computed in four age-groups: 0–19, 20–44, 45–64 and 65+.

The quantity dispensed was computed in terms of defined daily dose (DDD) per 1000 people per day unit. The DDD unit corresponds to the assumed average maintenance daily dose of the medicine for an adult when used for its main indication [18]. Average quantities in DDD/1000 people/day for major items of opioids and benzodiazepines dispensed in the individual years and states/territories was computed and graphed to compare the trends. As we used a 10% sample, the quantities were multiplied by 10 to have a population level estimate.

We used the generalized linear approach for developing statistical models where the dependent variable was user type, *namely* single-quarter user, medium-episodic user, long-episodic user, and chronic user. As the dataset was hierarchically structured (e.g., LGAs are nested in states), we performed multilevel mixed-effects generalized linear model using *meglm* command of the STATA program [19]. Independent variables used in the models were sex, age of the clients, socio-economic index of the locations of the patients, LGA urbanization status (urban or rural), dispensing of benzodiazepines/opioids during the study period, LGA (as level 2) and state/territory (as level 3). In the regression model for opioids, the history of benzodiazepines dispensing during the study period (yes or no) was used as an independent variable. Similarly, in the regression model for benzodiazepines, the history of opioids dispensing was used as an independent variable.

As the multilevel model for such a large dataset needs calculation of residuals at each iteration and hence a powerful computer with large memory, we took a random sample of 100,000 patients for developing this model. This study was approved by the La Trobe University Human Research Ethics Committee (approval: S17-003).

## Results

**Fig 1:**
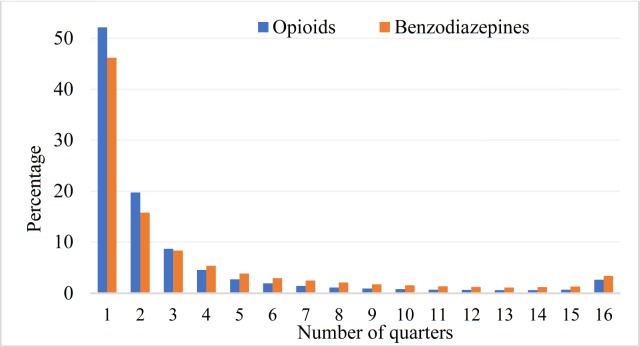
Distribution of users in terms of percentages and dispensing duration over the 16 quarters.

The sample had 795,578 clients, 46% were male and 54% female. The mean age of the overall sample participants was 50.6 years (SD ± 20.18), of male clients was 50.1 years and female clients was 51.1 years. Most (85%) were dispensed opioids and 41% were dispensed benzodiazepines and almost 26% were dispensed both medicines. The mean age of the clients who were dispensed opioids was 50.4 years, benzodiazepines was 54.5 years. Overall, around 52% of opioids users and 46% of benzodiazepines users were dispensed these medicines for a single quarter. Among those who were dispensed three or fewer quarters, a higher proportion received opioids than benzodiazepines (Fig 1). The single quarter and medium-episodic users were mainly of 20–44 years age-group, and this trend was consistent for opioids and benzodiazepines users. Conversely, the regular and long-episodic users were predominantly the senior citizens (age 65+ years). The proportions of long-episodic and chronic use gradually increased with the increasing age of the users.

By counting the individual calendar month to which dispensing was recorded for each client, the average for opioids users was found 4.65 months (SD± 8.64, range 1 to 48) and benzodiazepines users was found 5.64 months (SD± 8.99, range 1 to 48). On average and across the age-groups, benzodiazepines dispensing was recorded in a greater number of calendar months than opioids. The duration of dispensing recorded in terms of total number of quarters increased with age.

**Fig 2:**
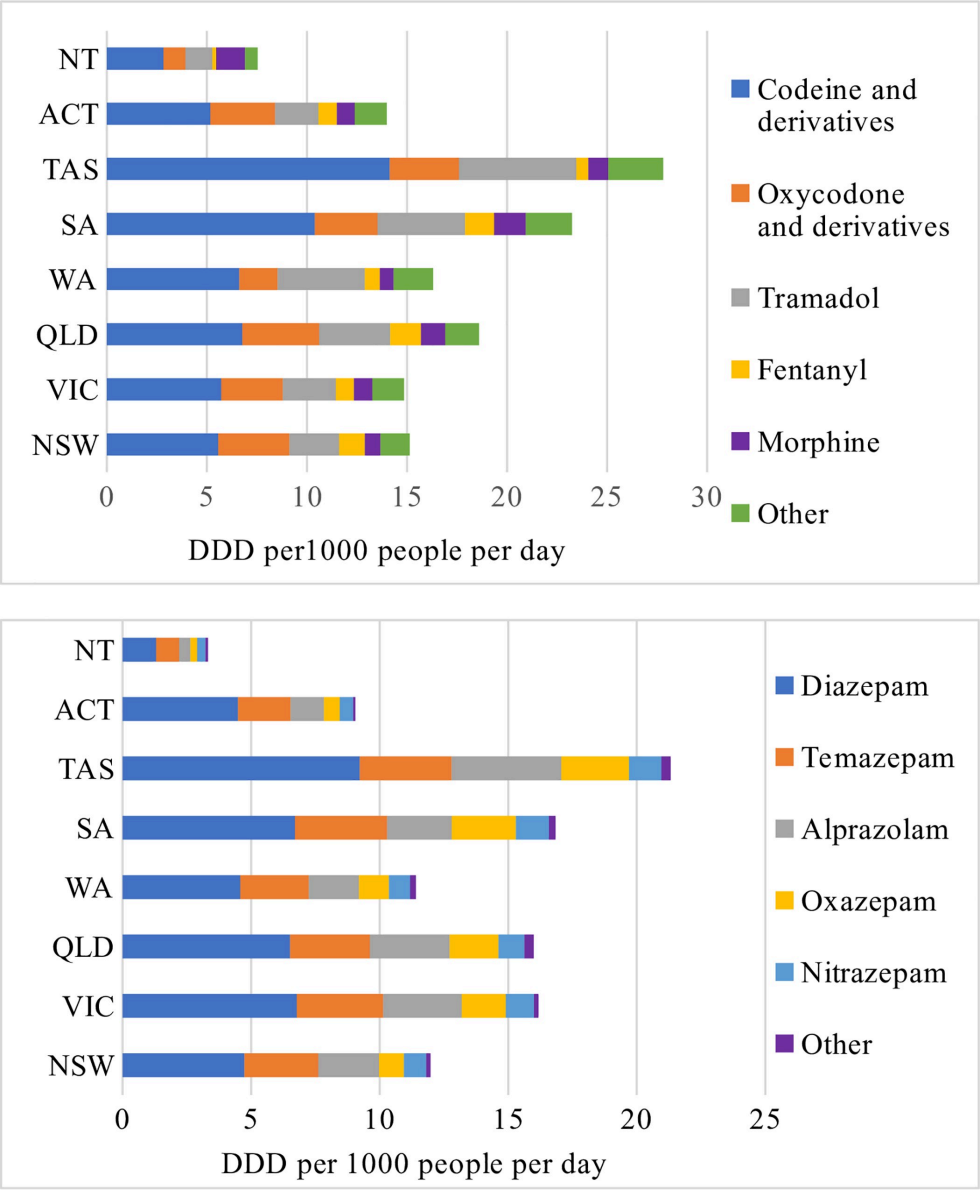
Dispensing of opioids and benzodiazepines in terms of DDD per 1000 people per day.

During the study period, on average, 16.6 DDD/1000 people/day of opioids and 14.2 DDD/1000 people/day of benzodiazepines were dispensed. Codeine (and derivatives) was the most prevalent item in all states and territories both in terms of number of users being dispensed and DDD/1000 people/day (Fig 2). Oxycodone was the second most dispensed item in terms of DDD/1000 people/day in all states/territories except Western Australia, South Australia, Tasmania, and Northern Territory. In these four jurisdictions, tramadol was dispensed more than oxycodone (and derivatives) (Fig 2). Tasmania was dispensed the highest quantity of opioids followed by South Australia, in terms of DDD/1000 people/day.

In almost all states/territories, diazepam was the most dispensed benzodiazepine items followed by temazepam and alprazolam (Fig 2). More alprazolam than temazepam was dispensed in Tasmania. Similar to opioids dispensing, Tasmania and South Australia were dispensed more quantities of benzodiazepines than any other states/territories. Dispensing in DDD/1000 people/day was higher among women than among men, both for opioids and benzodiazepines.

**Fig 3:**
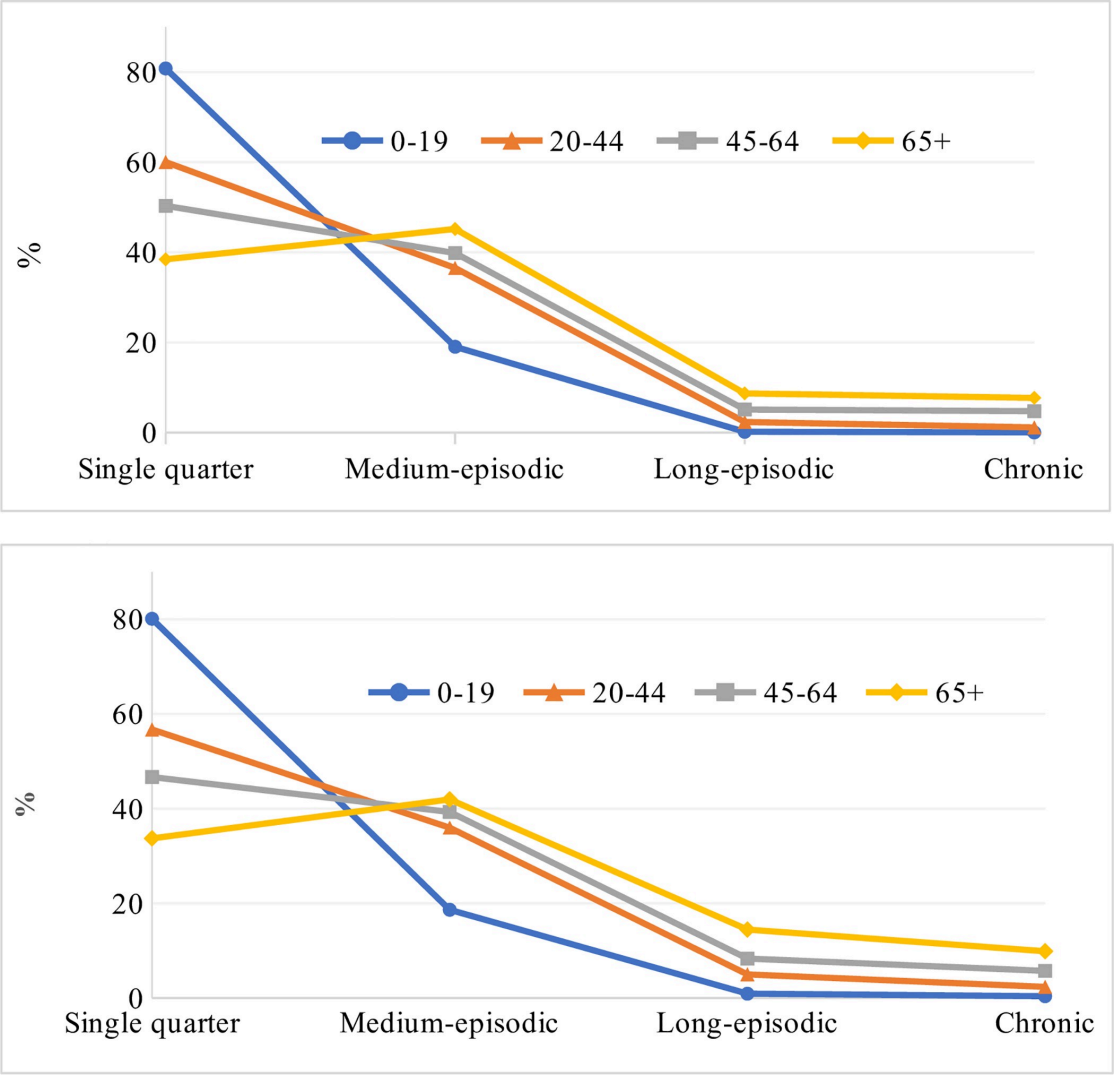
Four categories of dispensing of opioids and benzodiazepines across four age-groups.

Overall, 52% were single quarter, 39% medium-episodic, 5% long-episodic and 4% were chronic users of opioids. Among those who were dispensed benzodiazepines, 46% were single quarter, 39% medium-episodic, 9% long-episodic and 6% were chronic users. Single quarter users were the dominant group in all except the 65+ year age-group (Fig 3). There was a clear trend in age-groups with medium-episodic, long-episodic and chronic users. This trend was consistent both for opioids and benzodiazepines.

In terms of DDD/1000 people/day, around 60% of opioids were dispensed to the chronic users, 20% to the long-episodic, 16% to the medium-episodic and 4% to the single quarter users. These percentages for benzodiazepines dispensing were 50%, 25%, 21%, and 4%, respectively.

In comparison to men, women were significantly more likely to have been dispensed opioids or benzodiazepines for a relatively long duration (Table 1). Compared to those of age-group 0–19, clients of other age-groups (i.e., 20–44, 45–64 and 65+) were more likely to have dispensed opioids or benzodiazepine for a relatively long duration. There were gradients in the strength of associations across the age-groups, adjusted odds ratios (AORs) increased with age (Table 1). SEIFA was a significant factor in the model for opioids dispensing, AORs increased with levels of disadvantage. A similar association was found for the urbanization of the LGAs. The associations of SEIFA and urbanization in the model for benzodiazepines dispensing were not significant (Table 1). Those who were dispensed benzodiazepines during the study period were 2.52 times more likely than others to have dispensed opioids for a relatively long duration. On the other hand, those who were dispensed opioids were 1.51 times more likely than others to have dispensed benzodiazepines for a relatively long duration.

**Table 1:** Generalized multilevel ordinal models examining factors associated with dispensing duration of opioids and benzodiazepines recorded in terms of number of quarters.

Variable	Opioids (n = 83319)		Benzodiazepines (n = 40393)	
	AOR (95% CI)	*p*	AOR (95% CI)	*p*
**Sex**				
Male	1	-	1	-
Female	1.11 (1.08–1.14)	<0.01	1.17 (1.13–1.22)	<0.01
**Age**				
0–19	1	-	1	-
20–44	2.46 (2.26–2.67)	<0.01	3.60 (2.94–4.41)	<0.01
45–64	3.86 (3.55–4.20)	<0.01	5.60 (4.58–6.85)	<0.01
65+	6.34 (5.82–6.90)	<0.01	9.93 (8.11–12.15)	<0.01
**SEIFA**				
Very high	1	-	1	-
High	1.33 (1.26–1.41)	<0.01	0.99 (0.93–1.05)	0.78
Moderate	1.41 (1.32–1.50)	<0.01	1.00 (0.93–1.07)	0.98
Least	1.59 (1.48–1.71)	<0.01	1.01 (0.92–1.08)	0.97
**Urbanization**				
Urban	1	-	1	-
Rural	1.12 (1.05–1.19)	<0.01	0.95 (0.88–1.04)	0.27
**Dispensed benzodiazepines/opioids** ^**§**^				
No	1	-	1	-
Yes	2.52 (2.44–2.59)	<0.01	1.51 (1.45–1.57)	<0.01
States/territories	0.001 (0.0003–0.01)	-	0.004 (0.001–0.02)	-
LGA	0.02 (0.01–0.28)	-	0.10 (0.005–0.02)	-
LR test vs. negative binomial model: chi2(2)	156.89	<0.01	35.01	<0.01

Note: AOR, Adjusted Odds Ratio

^§ ^Dispensing of benzodiazepines were used when developing model for opioids and vice versa.

## Discussion

Analysis of this large dataset suggests while there was similarity in trends of dispensing of prescription opioids and benzodiazepines in Australia, there were substantial variations across jurisdictions. Both nationally and at individual states/territories, codeine (and derivatives) and diazepam were the most prevalent and dispensed items of opioids and benzodiazepines, respectively. Multilevel regression suggests women; those who were relatively old, living at rural areas or at locations of the relatively low socio-economic index, or dispensed benzodiazepines were more likely than others to have dispensed prescription opioids for a longer duration. Across the age-groups, benzodiazepines were found to have dispensed for a longer duration than opioids. Our findings also suggest that although 52% of opioid users and 46% of benzodiazepine users were dispensed these medicines for a single quarter, in terms of DDD/1000 people/day around 80% of opioids and 75% of benzodiazepines were dispensed among the chronic and long-episodic users. These observations, together, demand more careful prescribing and dispensing, particularly to the people who use for a longer duration, and for non-cancer pain. Also, as individuals with longer initial prescription periods are at risk of becoming long-episodic or chronic users [20], care is needed for these users as well.

Research on dispensing or utilization of these medicines indicates that greater use of prescription opioids or benzodiazepines does not necessarily translate into better health outcomes. In fact, high rates of use might produce worse outcomes such as risks for overdose and falls [21–23], and such use might both cause and be caused by misuse. Inappropriate use of these medicines occurs on a broad spectrum, ranging from those who unintentionally misuse them because of inappropriate prescribing practices through to those who intentionally obtain and misuse for their non-therapeutic effects (e.g., recreational use) and or for financial gain. Whether the prescription and dispensing of opioids or benzodiazepines was appropriate across jurisdictions was beyond the scope of this study. However, the adverse health outcomes from long-term use of these medicines, particularly when used concurrently, warrant the attention of both the prescribers and pharmacists. Recent data shows that although the proportion of concurrent benzodiazepine dispensing to opioid users in Australia has declined since 2012, it still remains relatively large (12.7%) [24].

Prescription opioids and benzodiazepines are useful medicines that offer a range of benefits. Literature suggests that most people use these medicines appropriately [25]. By analyzing the data of the latest round of the National Drug Strategy Household Survey, Chan and colleagues [26] found that in Australia the overall prevalence of past year non-medical pharmaceutical opioid use was 3.56%. Given that there is a demand for reducing untreated pain, and there exists a strong correlation between the quantity dispensed/the user numbers and the likelihood of inappropriate use, the public health measures mainly aim to reduce the inappropriate dispensing/utilization without hampering access for those who are duly in need to these medicines [27]. In the literature, a range of measures was described as effective [28,29] including real-time prescription drug monitoring program for prescribers and pharmacies [30,31]; awareness for patients and up-to-date training for prescribers and dispensers [32]; introduction and or expansion of drug treatment and or maintenance programs [33,34]; provision of alternative pain treatments [35,36]; and government legislation and enforcement [37,38].

There were substantial geographical variations in dispensing of prescription opioids and benzodiazepine across jurisdictions. These variations are likely to be attributed to a range of factors, some of which are structural, and some are related to the individual client. For instance, rurality-urbanity and SEIFA of the locations are structural and clients’ age and sex are individual characteristics. This wide range of factors suggests the importance of taking both short- and long-term programs. For some clients, inappropriate use of opioids and or benzodiazepines are just the outcome of deep-rooted social injustice and inequality and may warrant tackling social determinants of health [39]. A short-term program may not produce a sustainable solution to this subgroup. While tackling social determinants of health may sound like a distant solution, particularly with regard to our study findings, provision of a favorable set of social determinants and equity often produces benefits on several fronts and become long lasting and cost saving, when assessed from the societal perspective [40].

Factors that drive a relatively high-level dispensing of opioids and benzodiazepines in Tasmania are not completely understood. Misuse of these medicines by the illicit drug users may have partly contributed to this high-level dispensing. For instance, recent data suggests among all the states/territories, Tasmania (5.6%) and South Australia (5.5%) had the highest prevalence of pharmaceutical use for non-medical purposes [41]. Previous research also consistently found that relative to other jurisdictions, misuse of prescription opioids/benzodiazepines was more common in Tasmania. A relatively low supply of heroin and other illicit drugs to Tasmania may be one of the reasons for high demand for opioids/benzodiazepines [42,43]. Further research is needed to identify the extent to which this relatively high-level dispensing in Tasmania is attributable to illicit use and other factors such as relaxed prescribing or over-reliance on opioids and benzodiazepines.

Most states/territories in Australia have some levels of monitoring programs for clinicians and pharmacists, who have access to clients’ history of these medicines use, at real time, prior to prescribing and dispensing. Programs across the states/territories vary in approach and scale with relatively comprehensive programs active in some states such as Tasmania and the Australian Capital Territory. The outcome of the implementation of a real-time monitoring program in Tasmania appears to be generally positive. For instance, the number of deaths related to prescribed opioids reduced by a third to an average of 17 deaths a year between 2010 and 2014 [44]. Recently Victoria has introduced a real-time monitoring program in a small-scale. So far, the perception of this monitoring program is mostly positive [45,46]. However, there are many unanswered questions [47]. For instance, it is unclear as to how the legal ramifications will be implicated [48]. Also, variation across states/territories in approaches to day-to-day operation and monitoring should be taken into consideration and rectified as early as possible. We recommend the federal government to take proper initiatives to bring the consistency in utilization and reporting of drug monitoring programs so that data of one state/territory can be used by other states/territories and facilitate interstate communication and capacity to utilize these tools to their fullest potential.

Our results suggest a strong association between the dispensing of opioids and benzodiazepines. This observation is consistent with the finding of a study conducted in Norway that suggests an earlier use of benzodiazepines may predict repeated use of opioids. The use of benzodiazepines could be regarded as a proxy of psychiatric disorders (e.g., depression/anxiety), which was found to be a strong predictor of pain [49] and thus may cause initiation and continuation of opioid use. Also, pain can cause anxiety, depression, and insomnia and then use of benzodiazepines. Another possible reason is that often benzodiazepines users use these medicines for a relatively long-term [50]. As a result, timewise the longer the duration the more likely it is that the users will embark on other medicines including opioids. Also, the tendency of using benzodiazepines by those who use opioids for addiction, to some extent, may contribute to this observed association [51]. Before commencing treatment with opioids and or benzodiazepines, clinicians are recommended to balance the risk of problematic use of these drugs with the benefits [52].

One of the major strengths of this study is its large dataset. A 10% national sample of PBS, RPBS and under co-payment dispensing that constitute more than 80% of all dispensing [53] likely to have offered a representative and a reliable estimate. The study also has some limitations. The dataset did not capture opioids/benzodiazepines that were dispensed privately or from hospitals. Another limitation is that the study used dispensing but not utilization data. Although it is understandable that most of the dispensing is translated to utilization, little is known as to precisely how much is translated to the actual level of utilization. Also, this study finding does not inform as to whether and or to what extent the dispensing of these medicines was inappropriate for the individuals, across jurisdictions and nationally. Future research should aim to examine these. Besides, our results may not be generalizable to other country contexts. Finally, the way we categorized user-type may not be consistent with that in the literature. In fact, in the literature, there is no agreed taxonomy about this [54–56].

## Conclusions

There were similarities in terms of trends of dispensing of opioids and benzodiazepines in Australia. Overall, during the study period, more people were dispensed opioids than benzodiazepines. Those who were dispensed one of these two medicines were significantly more likely than others to have dispensed the other for a relatively long duration. People in socio-economically disadvantaged areas were more likely to use opioids for a relatively long duration and in high quantity. As the continuing use of these medicines has considerable side effects, and excessive use is a key public health concern, area-level research is recommended to explore the underlying factors that drive inappropriate prescribing and dispensing of opioids and benzodiazepines. Strict adherence to prescription/dispensing guidelines, provision of alternative treatments, consistent implementation of drug monitoring programs, and long-term structural interventions for tackling social determinants of health are recommended.
